# Clinical Uses Of Long-Duration Ultrasound And Long-Duration Sonophoresis In Sports Medicine - Minireview

**DOI:** 10.52338/tjop.2025.4566

**Published:** 2025-03-18

**Authors:** Rod Walters, David Snyder

**Affiliations:** 1 Consultants in Sports Medicine, Columbia, SC, USA; 2 Baylor University, Men’s Basketball, Waco, TC, US

**Keywords:** Sports Medicine, Long-Duration Ultrasound, Musculoskeletal pain, Sonoporation, Platelet-rich Plasma

## Abstract

Competitive physical sports demand rigorous training, increasing the risk of overuse-associated musculoskeletal traumatic injuries followed by a complex and time-consuming healing process with economic effects and potential disability. Tissue healing involves inflammation, molecular and cellular pathway regulation, proliferation, and tissue regeneration. These responses can significantly vary depending on the location and severity of the injury, affecting recovery time, pain intensity, range of motion, and return to sports activity. Despite medical advancements, healing, pain alleviation, regenerative tissue quality, mobility, and quality of life remain challenging. Current treatments, including nonsteroidal anti-inflammatory drugs and opioid-based treatments, have systemic adverse effects and efficacy limitations. Long-duration ultrasound therapy has emerged as a promising mechanobiological and diathermic treatment, providing critical biomechanical and thermal stimuli. Biomechanical stimuli help regulate acute inflammation, cellular proliferation, and tissue regeneration. Thermal stimuli enhance blood flow, angiogenesis, and nutrient exchange, accelerating healing and improving recovery outcomes. These combined stimuli also increase skin porosity and permeability, facilitating targeted drug delivery through sonoporation and enhancing the efficacy of treatments like platelet-rich plasma therapy. This review examines recent studies exploring the therapeutic effects of long-duration ultrasound as a standalone and adjunctive therapy. It examined its roles in regulating acute inflammation, mitigating chronic inflammation, tissue regeneration, healing, sports-associated pain management, mobility, and overall tissue recovery.

## INTRODUCTION

Healing of musculoskeletal (MSK) tissue is a complex physiological response regulated by tissue remodeling and regeneration following post-traumatic injury or extensive, intense physical training in sports. Tissue regeneration and remodeling is a highly regulated physiological process distributed over three main phases: an initial inflammatory phase, a tissue regeneration phase, and a remodeling phase(1). The length of healing and resultant tissue quality depends on several factors, including the nature and severity of the injury, the type of tissue affected, the overall health of the individual, and various physiological and genetic elements. The healing process can give rise to multiple complexities that may result in chronic inflammation, persistent pain, mechanically inferior tissue, low quality of life, and potential disability. This is further detrimental in sports, considering the extensive training and physical requirements. Conventional treatments such as nonsteroidal anti-inflammatory drugs (NSAIDs)(2), RICE therapy (Rest, Icing, Compression, and Elevation)(3), physical therapy, and rehabilitation are commonly employed to address both acute and chronic musculoskeletal (MSK) injuries(4). While these methods are effective, they possess limited long-term efficacy and potentially lead to adverse effects(2, 3, 4). Additionally, there has been medical advancement leading to therapies like platelet-rich plasma (PRP) therapy(5, 6), mesenchymal cell (MSC) therapy(7), low-level laser therapy (LLLT)(8), and extracorporeal shockwave therapy (ESWT)(9). These emerging treatments have shown promise in enhancing the healing and recovery process of MSK injuries, although their efficacy remains controversial(5, 6). Therefore, a significant number of patients still require surgery to enhance tissue healing and remodeling(10).

Ultrasound has proven to be an effective therapeutic modality in treating numerous conditions such as tumors, cardiovascular diseases, central nervous system disorders, musculoskeletal system injuries, and management of acute and chronic pain(11, 12). Ultrasound is an acoustic wave providing essential biomechanical stimulation to the targeted tissues to activate and regulate targeted molecules and cells to enhance and regulate cellular and molecular activity(12). Therapeutic ultrasound has a clinical history as a widely used modality to treat orthopedic and muscle injuries and pain management (13, 14). Ultrasound can have biological and biomechanical effects resulting in cellular regeneration(15), acute and chronic anti-inflammatory,(16) and thermal effects (17). Numerous studies have shown that the efficacy of ultrasound is dependent on different factors, including intensity, wavelength, power, and duration(18, 19).

Long-duration ultrasound (LDU) administered with wearable medical systems utilizes consistent acoustic mechanical waves that activate molecular and cellular pathways, promoting the healing cascade(20, 21, 22). By delivering prolonged mechanical stimulation, LDU enhances skin porosity, allowing for the transfer of small molecules through a process known as sonophoresis(11, 14). Biomechanical forces play an essential role in stimulating the matrix cells to increase the formation of extracellular matrix proteins and improve alignment, resulting in stronger tissue with greater mechanical strength(23). The presence of biomolecular components improves cellular proliferation, regulation of cytokines and chemokines, and growth factors and alleviates pain(24, 25). The application of continuous LDU over an extended period generates heat, which leads to an increase in blood circulation, oxygenation, exchange of nutrients, availability of growth factors, proteolytic enzymes, amino acids, and other vital components to regulate the inflammatory cells and matrix remodeling(17, 22, 26, 27, 28).

**Figure 1:**
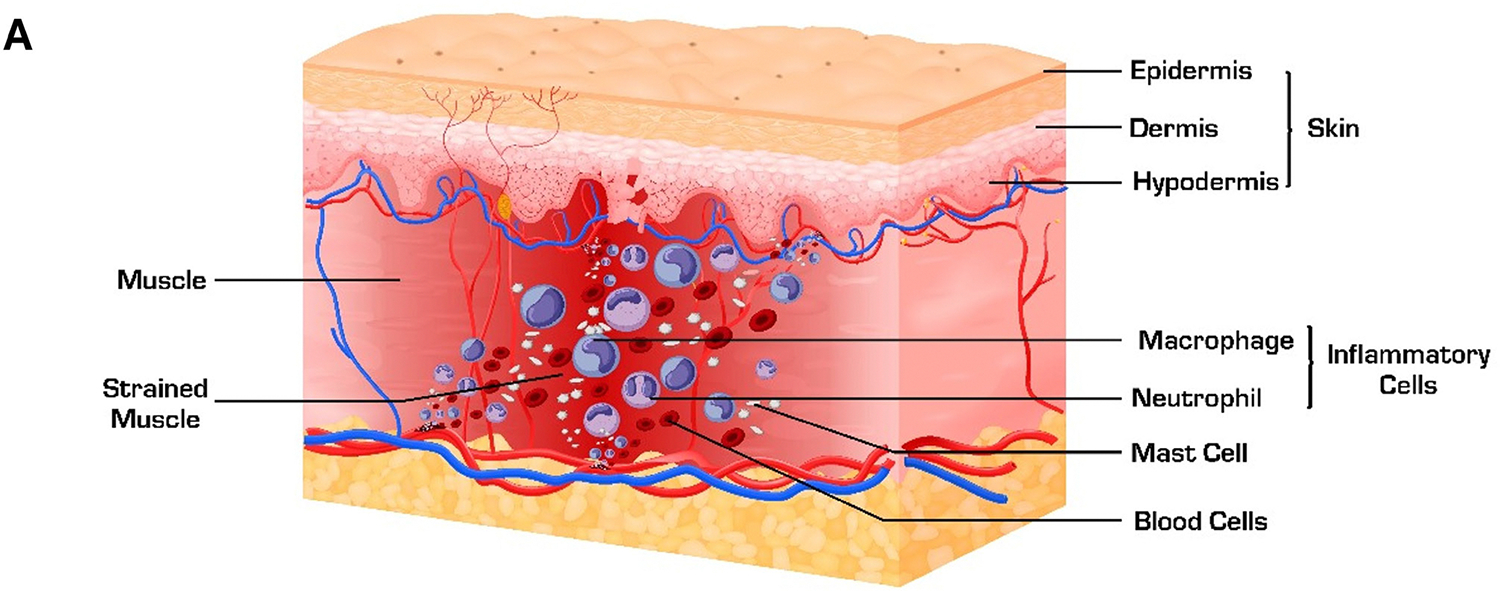

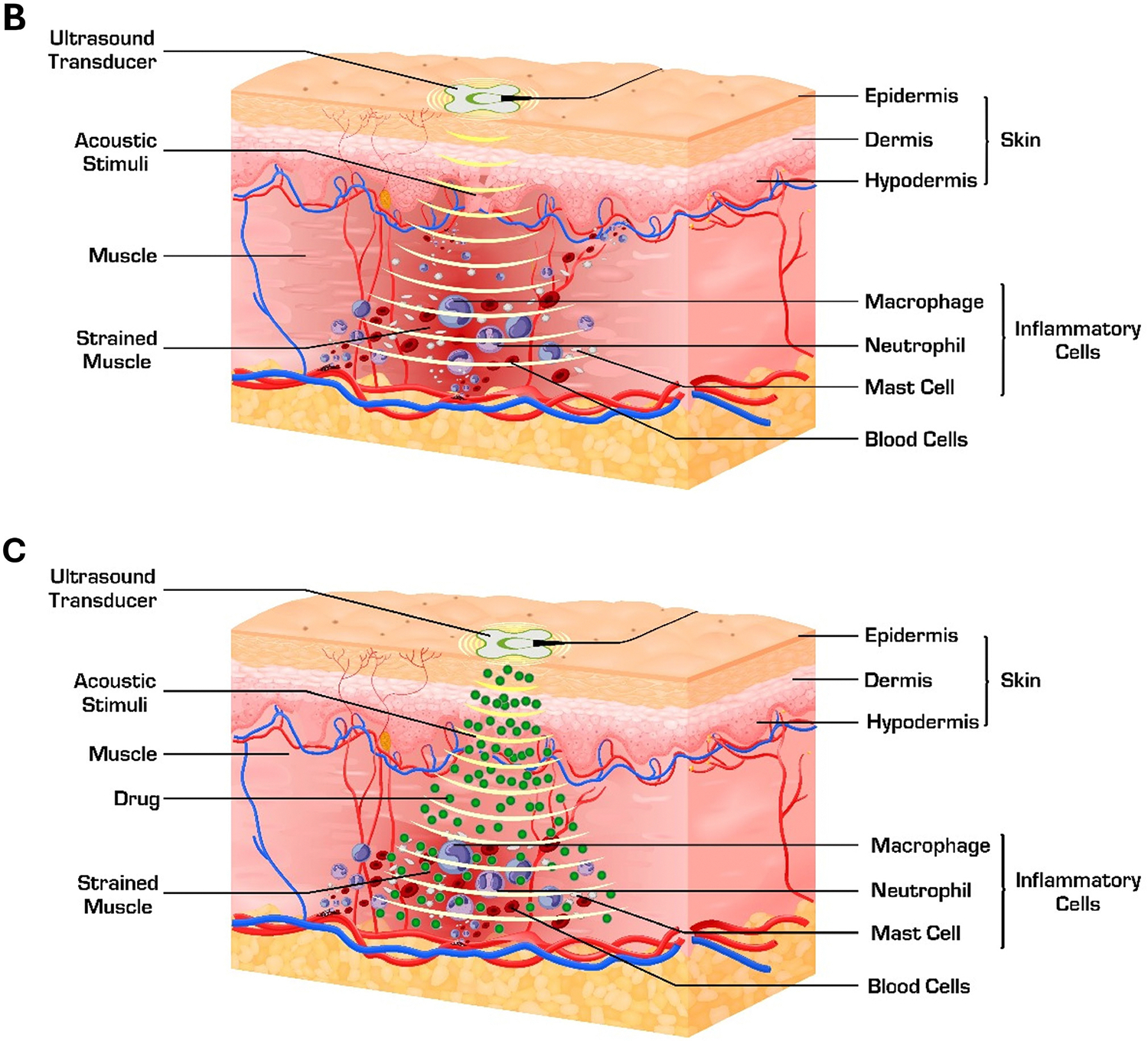
Synergistic Effects of Long Duration Ultrasound and Sonoporation. A) Inflammatory response to musculoskeletal injury. Marked muscle tissue damage with an increased level of inflammatory cells. B) LDU regulates the inflammatory response and improves tissue healing. C) The Synergistic effects of LDU/LDS enhance targeted drug delivery with accelerated tissue healing and regulate the inflammatory response.

Long-Duration Sonophoresis (LDS) increases the targeted drug delivery of drugs and small molecules through the skin and musculoskeletal tissues, enabling efficient and deeper drug delivery by utilizing mild mechanical energy over an extended period(14). The LDS in conjunction with LDU, have synergistic effects by providing biomechanical and biochemical components to accelerate the healing process by regulating inflammation, mitigating pain, and enhancing cellular proliferation, tissue regeneration, and remodeling(11, 14, 28). Figure 1, illustrates the impact of LDU as standalone therapy on acute inflammation and tissue healing and in combination with LDS as conjunctive therapy with synergistic effects in regulating inflammation and expediating tissue healing. Jarit et al. examined 135 patients suffering from musculoskeletal injuries and exhibited minimal or negligible improvement through conventional physical therapy. The application of LDS with 2.5% diclofenac shows a significant reduction in musculoskeletal injury pain when combined with physical therapy(29). The treatment alleviated the pain in soft tissues, joints, and bones, with the most notable improvements observed in the hip, lower back, and shoulder. The study shows a 2.5% diclofenac LDS as a promising treatment for patients with pain associated with musculoskeletal injuries when traditional bi-weekly physical therapy was not sufficient(29).

### THERMAL EFFECTS

**Figure 2:**
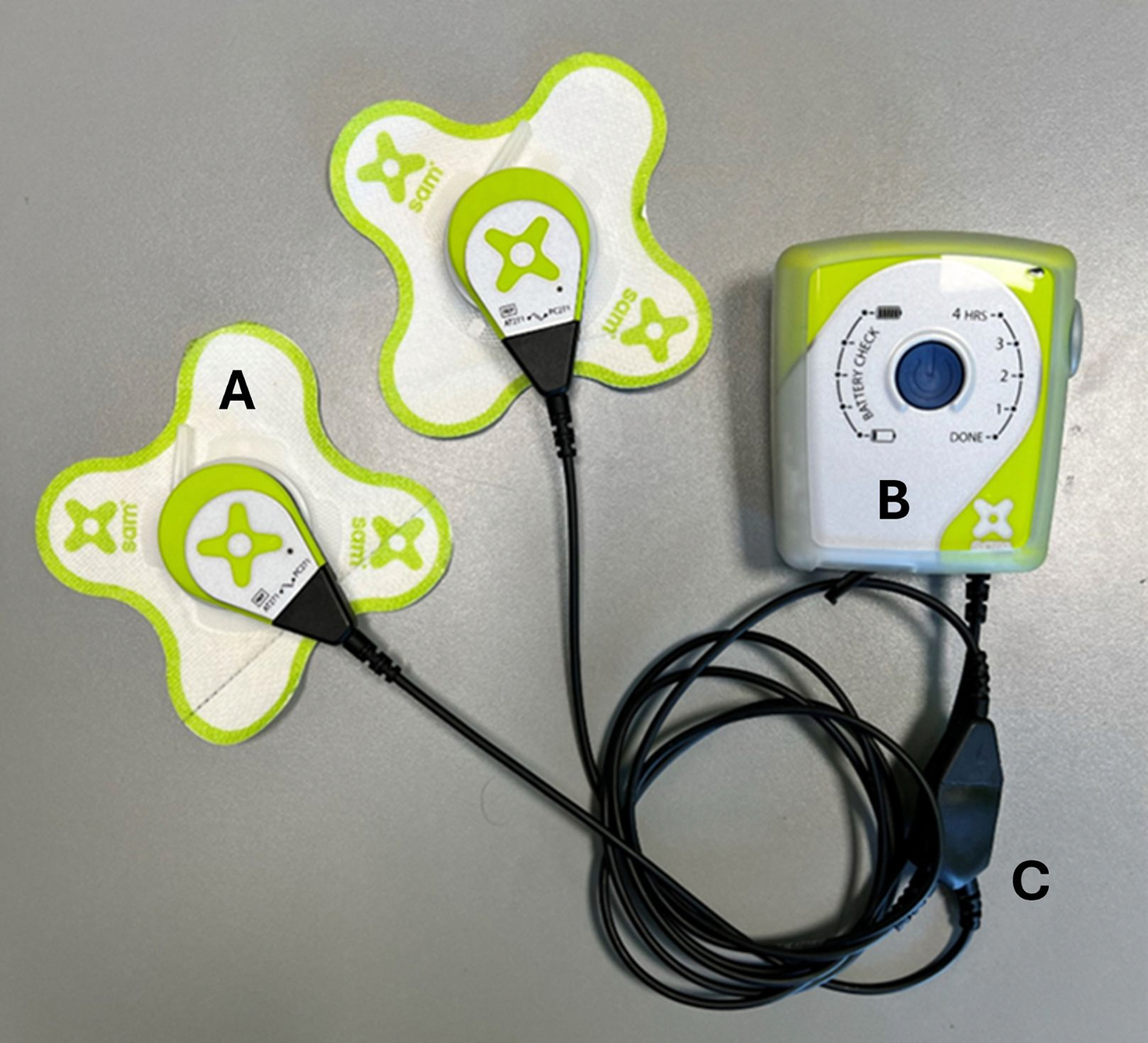
Basic 3 MHz LDU delivery system components for treating soft tissue injuries. A) Ultrasound transducer applicator and coupling patch, B) Ultrasound power controller and treatment timer, and C) Power splitter cable.

Single or multiple sessions of local heat therapy have been shown to increase the endothelial nitric oxide synthase (eNOS), a key regulator for vasomotor function and vascular remodeling. Increase in eNOS plays a vital role in increasing blood flow and angiogenesis, enhancing mitochondrial activity in skeletal tissue, and increasing glucose metabolism and insulin signaling(30). Draper et al. have argued that heat modalities like warm whirlpools, paraffin baths, and Hydrocollator HotPac^™^ have limited heat penetration depth (1cm) and only raise the temperature by 2°C in the human triceps muscle. Ultrasound therapy achieves a much more substantial effect by increasing temperature 6oC at a depth of 1 cm and by 4°C at a depth of 3cm (17, 31). Hayes et al. demonstrated that frequency plays a vital role, as 3-MHZ frequency is more efficient than 1-MHz in achieving greater thermal effects at a faster rate(31). 1 MHz is a lower frequency and will be absorbed slower as it penetrates through the skin into tissue relative to 3MHz, which has a higher energy density and absorption coefficient for thermal and biomechanical activity of LDU and LDS over time. Currently, 3 MHz is the primary carrier frequency for LDU systems approved for clinical use within the United States by the Food and Drug Administration(32)Figure 2 shows the basic components of a 3 MHz LDU and LDS delivery system, which includes a power controller with timer setting, ultrasound transducer head with energy director, and coupling patch to retain the couplet-gel and ultrasound transducer head in position for extended treatment.

Ultrasound-induced thermal effects increase blood circulation and oxygen dynamic by increasing localized levels of oxyhemoglobin(33). Elevating tissue temperatures by 2–4°C is vital for achieving therapeutic benefits. A moderate temperature rise of 2–3°C induces decreased muscle spasms and pain, increased blood flow, and reduced chronic inflammation(17, 34). To enhance viscoelastic alterations in collagen for improved stretching and joint mobilization, a temperature increase ≥ 4°C is necessary(34). Studies have shown against the previous belief that tendons heat up three times faster than muscle tissue when stimulated by 3MHz ultrasound due to the higher blood flow in muscle tissue having a more expanded vascular bed. Therefore, adjusting the intensity and duration of LDU is crucial to prevent tendons from overheating during the ultrasound treatment. Muscular recovery is quicker relative to tendons and ligaments which have less blood flow. Wearable technologies which deliver LDU as shown in Figure 2 have temperature regulation loops and keep tissue temperature around 41–44oC after 60 minutes or more of treatment; lower than the burning threshold.

Classically, therapist applied in-clinic ultrasound treatment for ten to twenty minutes typically delivers 700 to 2,000 Joules of energy deposition as a weekly rehabilitation approach for soft tissue injuries (35). LDU offers clinicians the opportunity to maximize ultrasound treatment by offering the extension of treatment away from the clinic, enabling more contact time both directly and indirectly with the patient. LDU systems are FDA-approved for home-use, and enable patients to self-apply non-invasive mechanobiological stimulation for up to four hours per day with total energy deposition greater than 18,000 Joules(36). LDU treatment for musculoskeletal injuries and disorders focuses on daily and longer treatment durations, increasing energy deposition and accelerate healing. LDU home-use systems produce ultrasound without pulses and increase muscle temperature, creating a potential for increased blood flow(19), increased connective tissue extensibility(37), altered nerve conduction velocity(38), and with less probability of forming adverse standing waves leading to potential tissue damage(39). Multiple studies suggest that daily increased energy deposition via LDU can improve a patient’s quality of life(40, 41). Uddin et al. reviewed recent literature on the efficacy of LDUs in decreasing pain and improving function in musculoskeletal injuries (13, 20, 21, 36). Petterson et al. determined the effectiveness of daily four-hour LDU at alleviating upper trapezius active myofascial pain and muscle tenderness over a 4-week treatment period. The study reported a significant pain reduction and health improvement from LDU compared to placebo treatment (42).

### SPORTS INJURY RECOVERY

In sports, overloading the musculoskeletal system can result in excessive wear and tear and fatigue, making athletes susceptible to injuries(43). This recovery from the injuries bears both physiological and psychological ramifications on athletes, requiring extensive training and exercise(43). Overuse can lead to compromised tissue that is not healed to pre-injury levels and is unable to sustain forces associated with the activity. To accelerate the recovery process, various modalities are utilized to enhance the regeneration of tissues, including cold-water immersion (CWI), massage therapy, and therapeutic rest(44, 45). Psychological recovery is attained through various strategies, including meditation, relaxation and sleep cycles, and nutrition. It should not be understated the importance of minimizing the use of systemic non-steroidal anti-inflammatory (NSAIDs) which are non-specific and can inhibit regular recovery of non-injured and injured tissues particularly for the avid athlete (46).

Sports Medicine research is vital in identifying new modalities to promote tissue regeneration and expedite recovery(44). LDU provides an innovative, non-invasive, and targeted approach to the recovery process by increasing the local temperature and mechanical stimulus, resulting in angiogenesis, inhibition of chronic inflammation, and driving cellular differentiation and proliferation, leading to tissue regeneration(16, 20, 36). Furthermore, LDS enhances targeted drug delivery, alleviating the pain associated with recovery and tissue regeneration processes at different phases of tissue regeneration(11, 14, 28). LDS is targeted, deep and specific to the injury site and therefore reduces the systemic consequences of oral NSAIDs or large surface application of topical therapeutics(28). In 2021, Draper et al. reported LDU as an easy-to-use, wearable, user-friendly, and comfortable “go-to” device outside the athletic training facility. Professional sports healthcare providers determined an 87% satisfaction and increased confidence in the ability of LDU to accelerate the healing process.

Data from three hundred and seventy-two patients were reviewed by Winkler et al., and results showed LDU is clinically effective in treating musculoskeletal injuries. Therefore, LDU was recommended by government healthcare providers of the USA Veterans healthcare system to use as an adjunct therapy with traditional treatment to accelerate recovery from multiple musculoskeletal injuries. Walters et al. conducted a comprehensive analysis, combining survey results and in-person evaluations of healthcare providers in professional, collegiate, and military medicine. The data demonstrated that LDU application in the treatment of sports medicine-related injuries provided both physical and mental benefits for patients. Notably, 83% of respondents reported that LDU effectively relieved pain in their patients(23). Importantly, the LDU application elevated the confidence of athletes and sports medicine staff in the recovery process. Overall, this led to accelerated recovery speed and enhanced strength while reducing reliance on oral pain medication. The outcomes yielded positive effects on both physical and psychological behavior.

### LDS USE POST-ACUTE IN INJURY

Sonoporation raises skin temperature, thereby increasing skin permeability. Numerous investigations have substantiated the efficacy of this approach in facilitating the delivery of drugs at low intensities through the skin (47). Cagnie et al. conducted a study revealing a remarkable tenfold escalation in ketoprofen concentrations within synovial tissue at 1 MHz and 1.5 W/cm² for a duration of 5 minutes compared to the conventional topical application of ketoprofen. Langer et al. employed a Long-Duration Sonophoresis (LDS) strategy to analyze the transdermal penetration of salicylic acid within a hydrogel system designed for in vitro drug delivery(48). Masterson et al. explored the penetration of diclofenac sodium through a hydrogel stack using LDS, observing a 3.8-fold increase in drug penetration through the hydrogel stack, mimicking an ex vivo transdermal model (14). Madzia et al. conducted a clinical study involving 32 patients with moderate to severe knee osteoarthritis (OA) subjected to LDU treatment for one week concurrently with applying sodium diclofenac patches(29). Following seven days of treatment, patients exhibited a noteworthy decrease in pain on the Numerical Rating Scale (NRS) by 2.06 to 2.96 points and a substantial improvement in functionality on the Western Ontario and McMaster Universities Osteoarthritis Index (WOMAC) scale by 351 to 510 points. These findings collectively underscore the potential therapeutic benefits of sonophoresis in enhancing drug delivery and mitigating symptoms associated with musculoskeletal conditions.

### PLATELET-RICH PLASMA INDICATIONS

Platelet-rich plasma, derived from the patient’s own blood, is rich in growth factors that play a key role in tissue repair. Meta-analysis by Chen et al. have reported positive outcomes when PRP is used in the treatment of ligament injuries, osteoarthritis, and other musculoskeletal conditions(49). Professional athletes have shown encouraging results utilizing PRP injection in treating musculoskeletal injuries, enhancing the healing process, and reducing the application of NSAIDs over time(5, 6). Krystofiak et al. combined PRP and LDU treatment to enhance pain relief, range of motion, and global health score and decreases the number of days to return to professional sports after soft tissue injury(50). The combined approach shows promise as an effective and innovative therapeutic strategy, but further research is needed to establish standardized protocols and guidelines for its implementation in sports medicine.

## CONCLUSION

Sports provide physical activity and substantial economic revenue, but this socioeconomic benefit has immense health cost. The excessive physical strain on the body leads to a higher prevalence of injuries, particularly MSK injuries. Over decades, multiple medical advancements have been made to enhance the complicated recovery process, alleviate injury-associated pain, improve resultant tissue quality, and reduce the number of days of return to active sports. Despite the medical advancement, a significant number of injuries require surgeries, further complicating the recovery process, increasing the number of days to return to active sports, and higher prevalence of future injury.

Long Duration Ultrasound (LDU) treatment provides acoustic biomedical and thermal stimulus enhancing temperature and local skin porosity. LDU promotes targeted drug delivery, increases blood circulation, tissue oxygenation, and nutrition exchange, and decreases inflammation. This expedites tissue recovery through cellular differentiation, proliferation, and extracellular matrix deposition, resulting in accelerated tissue regeneration and pain alleviation. Sports medicine providers recommend that patients actively utilize LDU in conjunction with conventional therapies, including NSAIDs, physical therapy, stem cells, and PRP treatments, to improve the patients’ quality of life, significantly expedite their return to activity, and reduce the overall recovery period.

## References

[1] Laumonier T, Menetrey J, Huard J. Basic Principles of Muscle Healing. In: Kerkhoffs GMMJ, Servien E, editors. Acute Muscle Injuries. Cham: Springer International Publishing; 2014. p. 17–26.

[2] AtchisonJW, HerndonCM, RusieE. NSAIDs for musculoskeletal pain management:current perspectives and novel strategies to improve safety. J Manag Care Pharm. 2013;19(9 Suppl A):S3–19.24261788

[3] van den Bekerom MP, Struijs PA, Blankevoort L, Welling L, van Dijk CN, Kerkhoffs GM. What is the evidence for rest, ice, compression, and elevation therapy in the treatment of ankle sprains in adults? J Athl Train. 2012;47(4):435–43. 22889660 10.4085/1062-6050-47.4.14 PMC3396304

[4] Crowell MS, Deyle GD, Owens J, Gill NW. Manual physical therapy combined with high-intensity functional rehabilitation for severe lower extremity musculoskeletal injuries: a case series. J Man Manip Ther. 2016;24(1):34–44. 27252581 10.1179/2042618614Y.0000000076 PMC4870039

[5] Sanchez M, Anitua E, Orive G, Mujika I, Andia I. Platelet-rich therapies in the treatment of orthopaedic sport injuries. Sports Med. 2009;39(5):345–54. 19402740 10.2165/00007256-200939050-00002

[6] Fang J, Wang X, Jiang W, Zhu Y, Hu Y, Zhao Y, Platelet-Rich Plasma Therapy in the Treatment of Diseases Associated with Orthopedic Injuries. Tissue Eng Part B Rev. 2020;26(6):571–85. 32380937 10.1089/ten.teb.2019.0292 PMC9208862

[7] Torres-Torrillas M, Rubio M, Damia E, Cuervo B, Del Romero A, Pelaez P, Adipose-Derived Mesenchymal Stem Cells: A Promising Tool in the Treatment of Musculoskeletal Diseases. Int J Mol Sci. 2019;20(12). 10.3390/ijms20123105 PMC6627452 31242644

[8] Doyle AT, Lauber C, Sabine K. The Effects of Low-Level Laser Therapy on Pain Associated With Tendinopathy: A Critically Appraised Topic. J Sport Rehabil. 2016;25(1):83–90. 25559198 10.1123/jsr.2014-0219

[9] Schroeder AN, Tenforde AS, Jelsing EJ. Extracorporeal Shockwave Therapy in the Management of Sports Medicine Injuries. Curr Sports Med Rep. 2021;20(6):298–305. 34099607 10.1249/JSR.0000000000000851

[10] Stinner DJ, Edwards D. Surgical Management of Musculoskeletal Trauma. Surg Clin North Am. 2017;97(5):1119–31. 28958361 10.1016/j.suc.2017.06.005

[11] Yang C, Li Y, Du M, Chen Z. Recent advances in ultrasound-triggered therapy. J Drug Target. 2019;27(1):33–50. 29659307 10.1080/1061186X.2018.1464012

[12] Uddin SMZ, Komatsu DE. Therapeutic Potential Low-Intensity Pulsed Ultrasound for Osteoarthritis: Preclinical and Clinical Perspectives. Ultrasound Med Biol. 2020;46(4):909–20. 31959508 10.1016/j.ultrasmedbio.2019.12.007

[13] Chung JI, Min BH, Baik EJ. Effect of Continuous-Wave Low-Intensity Ultrasound in Inflammatory Resolution of Arthritis-Associated Synovitis. Phys Ther. 2016;96(6):808–17. 26586863 10.2522/ptj.20140559

[14] Masterson J, Kluge B, Burdette A, Sr GL. Sustained acoustic medicine; sonophoresis for nonsteroidal anti-inflammatory drug delivery in arthritis. Ther Deliv. 2020;11(6):363–72. 32657251 10.4155/tde-2020-0009 PMC7373207

[15] Best TM, Wilk KE, Moorman CT, Draper DO. Low Intensity Ultrasound for Promoting Soft Tissue Healing: A Systematic Review of the Literature and Medical Technology. Intern Med Rev (Wash D C). 2016;2(11). 10.18103/imr.v2i11.271 PMC6128661 30198009

[16] da Silva Junior EM, Mesquita-Ferrari RA, Franca CM, Andreo L, Bussadori SK, Fernandes KPS. Modulating effect of low intensity pulsed ultrasound on the phenotype of inflammatory cells. Biomed Pharmacother. 2017;96:1147–53. 29191696 10.1016/j.biopha.2017.11.108

[17] Draper DO, Castel JC, Castel D. Rate of temperature increase in human muscle during 1 MHz and 3 MHz continuous ultrasound. J Orthop Sports Phys Ther. 1995;22(4):142–50. 8535471 10.2519/jospt.1995.22.4.142

[18] Feng F, Mal A, Kabo M, Wang JC, Bar-Cohen Y. The mechanical and thermal effects of focused ultrasound in a model biological material. J Acoust Soc Am. 2005;117(4 Pt 1):2347–55. 15898675 10.1121/1.1873372

[19] Hauck M, Noronha Martins C, Borges Moraes M, Aikawa P, da Silva Paulitsch F, Mea Plentz RD, Comparison of the effects of 1MHz and 3MHz therapeutic ultrasound on endothelium-dependent vasodilation of humans: a randomised clinical trial. Physiotherapy. 2019;105(1):120–5. 29373113 10.1016/j.physio.2017.08.010

[20] Uddin SMZ, Komatsu DE, Motyka T, Petterson S. Low-Intensity Continuous Ultrasound Therapies-A Systematic Review of Current State-of-the-Art and Future Perspectives. J Clin Med. 2021;10(12). 10.3390/jcm10122698 PMC8235587 34207333

[21] Best TM, Moore B, Jarit P, Moorman CT, Lewis GK. Sustained acoustic medicine: wearable, long duration ultrasonic therapy for the treatment of tendinopathy. Phys Sportsmed. 2015;43(4):366–74. 26468991 10.1080/00913847.2015.1095617

[22] Walters R, Kasik J, Ettel C, Ortiz R. Evaluation of Sustained Acoustic Medicine for Treating Musculoskeletal Injuries in Military and Sports Medicine. Open Orthop J. 2022;16. 10.2174/18743250-v16-e221130-2022-8 PMC9869494 36694709

[23] Chen M, Guo W, Gao S, Hao C, Shen S, Zhang Z, Biomechanical Stimulus Based Strategies for Meniscus Tissue Engineering and Regeneration. Tissue Eng Part B Rev. 2018;24(5):392–402. 29897012 10.1089/ten.TEB.2017.0508

[24] Atcha H, Jairaman A, Holt JR, Meli VS, Nagalla RR, Veerasubramanian PK, Mechanically activated ion channel Piezo1 modulates macrophage polarization and stiffness sensing. Nat Commun. 2021;12(1):3256. 34059671 10.1038/s41467-021-23482-5 PMC8167181

[25] Bakowski P, Mieloch AA, Porzucek F, Mankowska M, Ciemieniewska-Gorzela K, Naczk J, Meniscus repair via collagen matrix wrapping and bone marrow injection: clinical and biomolecular study. Int Orthop. 2023. 10.1007/s00264-023-05711-2 PMC10522727 36764942

[26] Petterson S, Plancher K, Klyve D, Draper D, Ortiz R. Low-Intensity Continuous Ultrasound for the Symptomatic Treatment of Upper Shoulder and Neck Pain: A Randomized, Double-Blind Placebo-Controlled Clinical Trial. J Pain Res. 2020;13:1277–87. 32606899 10.2147/JPR.S247463 PMC7287226

[27] Rigby JH, Taggart RM, Stratton KL, Lewis GK Jr., Draper DO. Intramuscular Heating Characteristics of Multihour Low-Intensity Therapeutic Ultrasound. J Athl Train. 2015;50(11):1158–64. 26509683 10.4085/1062-6050-50.11.03 PMC4732395

[28] Jarit P, Klyve D, Walters R. Long Duration Sonophoresis of Diclofenac to Augment Rehabilitation of Common Musculoskeletal Injuries. Glob J Orthop Res. 2023;4(2). PMC9977165 36865667

[29] Madzia A, Agrawal C, Jarit P, Petterson S, Plancher K, Ortiz R. Sustained Acoustic Medicine Combined with A Diclofenac Ultrasound Coupling Patch for the Rapid Symptomatic Relief of Knee Osteoarthritis: Multi-Site Clinical Efficacy Study. Open Orthop J. 2020;14:176–85. 33408796 10.2174/1874325002014010176 PMC7784557

[30] Gibson OR, Astin R, Puthucheary Z, Yadav S, Preston S, Gavins FNE, Skeletal muscle angiogenic, regulatory, and heat shock protein responses to prolonged passive hyperthermia of the human lower limb. Am J Physiol Regul Integr Comp Physiol. 2023;324(1):R1–R14. 36409025 10.1152/ajpregu.00320.2021

[31] Hayes BT, Merrick MA, Sandrey MA, Cordova ML. Three-MHz Ultrasound Heats Deeper Into the Tissues Than Originally Theorized. J Athl Train. 2004;39(3):230–4. 15496991 PMC522144

[32] Lewis S Stationary ultrasonic diathermy device for use in applying therapeutic deep heat. In: Medicine MDP, editor. 2023.

[33] Morishita K, Karasuno H, Yokoi Y, Morozumi K, Ogihara H, Ito T, Effects of therapeutic ultrasound on intramuscular blood circulation and oxygen dynamics. J Jpn Phys Ther Assoc. 2014;17(1):1–7. 25792902 10.1298/jjpta.Vol17_001 PMC4316550

[34] Merrick MA, Bernard KD, Devor ST, Williams MJ. Identical 3-MHz ultrasound treatments with different devices produce different intramuscular temperatures. J Orthop Sports Phys Ther. 2003;33(7):379–85. 12918863 10.2519/jospt.2003.33.7.379

[35] Draper DO. A Comparison of Traditional Ultrasound and Sustained Acoustic Medicine (SAM). Orthopedics and Sports Medicine: Open Access Journal. 2019:2.

[36] Langer MD, Lewis GK, Jr. Sustained Acoustic Medicine: A Novel Long Duration Approach to Biomodulation Utilizing Low Intensity Therapeutic Ultrasound. Proc SPIE Int Soc Opt Eng. 2015;9467. 10.1117/12.2178213 PMC6070146 30078928

[37] Karnes JL, Burton HW. Continuous therapeutic ultrasound accelerates repair of contraction-induced skeletal muscle damage in rats. Arch Phys Med Rehabil. 2002;83(1):1–4. 11782824 10.1053/apmr.2002.26254

[38] Kramer JF. Effect of therapeutic ultrasound intensity on subcutaneous tissue temperature and ulnar nerve conduction velocity. Am J Phys Med. 1985;64(1):1–9. 3970155

[39] Secomski W, Bilmin K, Kujawska T, Nowicki A, Grieb P, Lewin PA. In vitro ultrasound experiments: Standing wave and multiple reflections influence on the outcome. Ultrasonics. 2017;77:203–13. 28254565 10.1016/j.ultras.2017.02.008 PMC5503701

[40] Draper DO, Klyve D, Ortiz R, Best TM. Effect of low-intensity long-duration ultrasound on the symptomatic relief of knee osteoarthritis: a randomized, placebo-controlled double-blind study. J Orthop Surg Res. 2018;13(1):257. 30326947 10.1186/s13018-018-0965-0 PMC6192104

[41] Zhou XY, Zhang XX, Yu GY, Zhang ZC, Wang F, Yang YL, Effects of Low-Intensity Pulsed Ultrasound on Knee Osteoarthritis: A Meta-Analysis of Randomized Clinical Trials. Biomed Res Int. 2018;2018:7469197. 30105243 10.1155/2018/7469197 PMC6076961

[42] Draper D, Mallipudi RM. Therapeutic ultrasound: myths and truths for non-portable in-clinic and portable home use ultrasound. MOJ Sports Med. 2020;4(4):115–6. 33442669 PMC7802764

[43] Halson SL. Monitoring training load to understand fatigue in athletes. Sports Med. 2014;44 Suppl 2(Suppl 2):S139–47. 25200666 10.1007/s40279-014-0253-z PMC4213373

[44] Kellmann M, Bertollo M, Bosquet L, Brink M, Coutts AJ, Duffield R, Recovery and Performance in Sport: Consensus Statement. Int J Sports Physiol Perform. 2018;13(2):240–5. 29345524 10.1123/ijspp.2017-0759

[45] Nedelec M, McCall A, Carling C, Legall F, Berthoin S, Dupont G. Recovery in soccer: part I - post-match fatigue and time course of recovery. Sports Med. 2012;42(12):997–1015. 23046224 10.2165/11635270-000000000-00000

[46] Roth SH, Fuller P. Diclofenac topical solution compared with oral diclofenac: a pooled safety analysis. J Pain Res. 2011;4:159–67. 21811391 10.2147/JPR.S20965 PMC3141832

[47] Carpentier A, Canney M, Vignot A, Reina V, Beccaria K, Horodyckid C, Clinical trial of blood-brain barrier disruption by pulsed ultrasound. Sci Transl Med. 2016;8(343):343re2. 10.1126/scitranslmed.aaf6086 27306666

[48] Matt Langer SL, Fleshman Shane, and Lewis George, editor “SonoBandage” a transdermal ultrasound drug delivery system for peripheral neuropathy. Proceedings of Meetings on Acoustics; 2013 17 May2013.

[49] Chen X, Jones IA, Park C, Vangsness CT Jr. The Efficacy of Platelet-Rich Plasma on Tendon and Ligament Healing: A Systematic Review and Meta-analysis With Bias Assessment. Am J Sports Med. 2018;46(8):2020–32. 29268037 10.1177/0363546517743746 PMC6339617

[50] Jason Krystofiak JB, Bates Ethan and Kummer Josh. Long Duration Ultrasound Combined with Platelet-Rich Plasma Injection for Return to Sport after Soft Tissue Injury: A Single Center Study. Orthopedic & Muscular System: Current Research. 2023;12(3):8. PMC10783208 38213829

